# Structural Retinal Analysis in Toxoplasmic Retinochoroiditis: OCT Follow-Up with Three-Dimensional Reconstruction

**DOI:** 10.3390/diagnostics15233091

**Published:** 2025-12-04

**Authors:** Ioana Damian, Adrian Pop, Adrian Groza, Elisabetta Miserocchi, Simona Delia Nicoară

**Affiliations:** 1 Department of Ophthalmology, “Iuliu Hațieganu” University of Medicine and Pharmacy, 8 Victor Babeș Street, 400012 Cluj-Napoca, Romania; 2 Clinic of Ophthalmology, Emergency County Hospital, 3-5 Clinicilor Street, 400006 Cluj-Napoca, Romania; 3 Artificial Intelligence Research Institute AIRi@UTCN, Technical University of Cluj-Napoca, 400114 Cluj-Napoca, Romania; 4 Department of Ophthalmology, IRCCS San Raffaele Scientific Institute, 20132 Milan, Italy; 5 Department of Ophthalmology, Vita-Salute San Raffaele University, 20132 Milan, Italy

**Keywords:** toxoplasmic retinal choroiditis, optical coherence tomography, three-dimensional reconstruction

## Abstract

**Background**: Ocular toxoplasmosis remains the leading cause of posterior uveitis worldwide. Optical coherence tomography (OCT) provides valuable insights into the structural alterations associated with this condition. The present study aimed to characterize the vitreous, retinal, and choroidal morphological changes observed during both the active and scarred stages of ocular toxoplasmosis using OCT imaging. A secondary objective was to evaluate the added value of three-dimensional reconstruction in the assessment of retinal lesions. **Methods**: A retrospective study was conducted on 12 eyes belonging to 12 patients diagnosed with toxoplasmosis retinochoroiditis (TRC). Optical coherence tomography (OCT) scans centered on the active lesions were qualitatively analyzed at baseline and follow-up. Additionally, a ResUNet model was trained to generate a full volumetric reconstruction of the retinochoroidal lesions in selected cases. **Results**: Twelve eyes were analyzed at a mean of 16.2 days from symptom onset. The mean follow-up duration was 144 days (range: 12–490 days). OCT imaging revealed characteristic alterations in the retina, choroid, and vitreous body, which were documented both at baseline and at follow-up. Representative cases were selected for three-dimensional reconstruction to illustrate the extent of retinal architectural involvement. **Conclusions**: OCT analysis refines our understanding of the structural damage associated with ocular toxoplasmosis, while three-dimensional reconstruction enhances our ability to visualize and interpret these alterations on a larger scale.

## 1. Introduction

Ocular toxoplasmosis is a parasitic infection of the retina and choroid caused by *Toxoplasma gondii*, and represents the leading cause of posterior uveitis worldwide. About 25–30% of the global population is systemically infected, making it the most common foodborne parasitic disease [1]. Most infected individuals are asymptomatic or develop nonspecific symptoms such as fever or fatigue. Prevalence varies by region: low in Northern Europe, Southeast Asia, and North America (10–30%), moderate in Central and Southern Europe (30–50%), and very high in Latin America and tropical areas, with Brazil reporting the highest rates [1]. Cats are the definitive hosts, while humans and other mammals are intermediate hosts. Transmission occurs through ingestion of oocysts from contaminated food or water, or tissue cysts from undercooked meat [2]. Infection may be congenital (via placental transmission) or acquired (usually gastrointestinal). Postnatal infection is more frequent, accounting for two-thirds of cases [3]. The possibility of sexual transmission of toxoplasmosis has been documented in animals, while in humans *T. gondii* has been detected in semen.

Ocular involvement primarily affects the retina, with secondary extension to the choroid, vitreous, and anterior chamber [4]. It usually manifests as progressive necrotizing retinitis, with complications such as iridocyclitis, retinal detachment, choroidal neovascularization, cataract, glaucoma, macular edema, or optic nerve atrophy. Atypical forms such as neuroretinitis, occlusive vasculitis, punctate outer retinal toxoplasmosis, and pigmentary retinopathy are more common in immunocompromised patients [1,4]. Both parasite-specific and host-specific factors contribute to variable ocular manifestations [5]. Lymphadenopathy of the head and neck is a common sign in toxoplasmosis, usually soft, movable and sometimes painful.

The classic presentation is a satellite lesion: active retinochoroiditis adjacent to a chorioretinal scar. Diagnosis is mainly clinical, as seropositivity is common; IgM and IgG appear within 1–2 weeks of infection, with IgM disappearing within 6–9 months. Nonreactive IgG usually excludes disease in immunocompetent patients [2]. The acute phase lasts approximately 6 weeks, followed by scarring.

The disease is often self-limiting in immunocompetent patients, but therapy is recommended for vision-threatening or severe cases. Standard regimens combine antiparasitic agents with corticosteroids, most commonly pyrimethamine + sulfadiazine + steroids. Alternatives include clindamycin + sulfadiazine + steroids, or trimethoprim-sulfamethoxazole + steroids, with similar efficacy; lesion size is the main predictor of inflammation duration [6]. Treatment typically lasts 4–6 weeks. If systemic therapy is contraindicated, intravitreal clindamycin may be used. Corticosteroids are crucial to reduce inflammation and limit immune-mediated retinal damage.

The diagnosis of toxoplasmic retinochoroiditis is primarily clinical, and in atypical cases the diagnosis can be confirmed by polymerase chain reaction analysis of intraocular fluids.

However, ocular imaging such as OCT can be helpful in driving to the specific and correct diagnosis of ocular toxoplasmosis. OCT may reveal either active or scarred lesions, with each stage showing distinct structural characteristics. In cases of vitritis and poor examination conditions, sometimes OCT allows visualization of retinal lesions. Several clinical studies have analyzed OCT changes in active or scarred forms [4,7,8,9,10,11], but to our knowledge, these aspects have not been demonstrated using three-dimensional reconstruction.

The aim of the study was to describe vitreous, retinal, and choroidal morphological changes in the active and subsequently the scarred stages using OCT. Another objective was to assess the contribution of 3D reconstruction in evaluating the retinal lesion.

## 2. Materials and Methods

### 2.1. Case Selection

A retrospective analysis of 12 patients diagnosed with toxoplasmosis retinal choroiditis (TRC) was conducted. All patients were examined between 2017 and 2025 at the Uveitis Department of the Vita-Salute San Raffaele University Hospital, Milano, Italy, a tertiary center for uveitic patients.

### 2.2. Diagnosis

All patients underwent a comprehensive ophthalmologic evaluation, including best-corrected visual acuity (BCVA), slit-lamp examination, and fundus examination at baseline and follow-up visits. Active TRC was defined as the presence of a retinochoroidal lesion suggestive of *T. gondii*, with serological testing immunoglobulin G (IgG) and/or immunoglobulin M (IgM) performed in only a few cases, as the clinical presentation was considered sufficiently characteristic. In atypical cases, ocular fluid was tested for *T. gondii* DNA using polymerase chain reaction (PCR).

Inclusion criteria were patients with clinically diagnosed retinochoroiditis and available OCT volumetric scans of the lesion both before and after treatment. Initially, eighteen patients were enrolled; however, six were excluded due to missing pre- and post-treatment OCT images.

### 2.3. Optical Coherence Tomography (OCT) Analysis

Spectral-domain OCT (SD-OCT) was performed with the Spectralis HRA + OCT system (Heidelberg Engineering, Heidelberg, Germany, software version 1.10.4.0). An SD-OCT scan was considered gradable if media clarity was sufficient to permit analysis of the retina and vitreoretinal interface.

OCT scans were centered on active lesions identified during clinical examination. These scans were obtained using a horizontal high-speed raster protocol of 30° × 30° area, composed of either 19 B-scans with ART (Automatic Real-Time Tracking) averaging 9 images separated by 120 μm, 97 B-scans with ART averaging 5 images separated by 60 μm, 49 B-scans with ART averaging 18 images separated by 30 μm, 61 B-scans with ART averaging 10 images separated by 120 μm, 25 B-scans with ART averaging 9 images separated by 20 μm, or 121 B-scans with ART averaging 5 images separated by 60 μm. The type of scan was selected by the physician based on media transparency and the ease of OCT image acquisition. All OCT scans intersecting the TRC lesion were qualitatively assessed by one of the authors (I.D.) to identify biomarkers characteristic of the active and healing phases. Retinal thickness was measured with Fiji software (version: 1.54p) calipers, defined as the distance from the internal limiting membrane to the basal RPE at the center of the lesion [12]. Choroidal thickness beneath active and scarred lesions was measured as the perpendicular distance from the outer border of the hyperreflective RPE to the choroidoscleral interface.

### 2.4. Volume Reconstruction Technique

**Figure 1:**
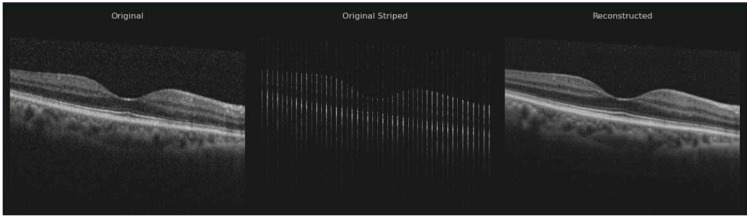
Original—true B-scan image; Original Stripped—synthetic stripped image for training; Reconstructed—result after inference.

Based on the assumption that the region scanned by OCT generally maintains consistent structural features across both its width and depth, we evaluated a ResUNet model designed to reconstruct the entire volumetric dataset from partial lateral scans. Specifically, given an incomplete lateral view, the model was trained to fill in the missing columns that result from the spacing between consecutive B-scans (Figure 1).

We collected OCT data from 12 patients grouped in 26 OCT scans, each containing 19, 25, 49, 97, 61, 73, or 121 B-scans, resulting in a total of 883 B-scans acquired using the Heidelberg Spectralis 2 system. Among these, 347 B-scans from 9 OCT scans were obtained using the High-Speed acquisition protocol and 536 B-scans from 6 OCT scans using the High-Resolution protocol. Following a visual quality assessment, 10 OCT volumes (corresponding to 427 B-scans) were excluded due to excessive image noise, misalignment, or axial or curvature deformation, as their correction would have required additional processing beyond the scope of this study. In these cases, the denoising algorithm was unable to reliably distinguish between speckle noise and retinal layers, resulting in structural alteration of the tissue boundaries.

**Figure 2:**
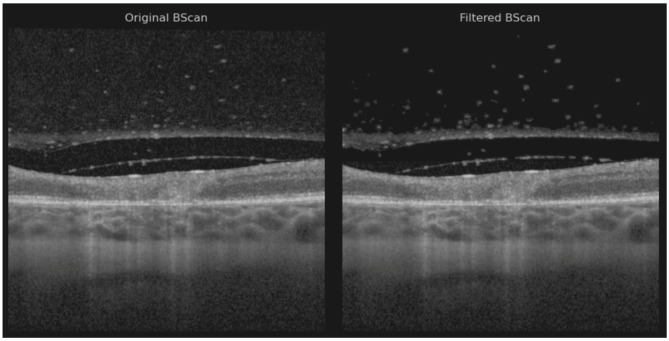
Original B-scan and filtered B-scan.

Each OCT volume was manually preprocessed to reduce speckle noise in the vitreous region while preserving small floating artifacts within the vitreous body (Figure 2) [13].

Noise reduction was performed using a hybrid approach that combined the pixel neighborhood structure with intensity-based object segmentation, as described by Babin et al. [14]—a method previously applied with success to isolate neovascular blood vessels in age-related macular degeneration (AMD) [15]. Two volumes were preprocessed using the layer segmentation tool before applying noise reduction due to the poor retinal pixel structure [16]. The vitreous artifacts may appear larger than usual because the noise reduction filter was not optimally sharp and required manual adjustment.

To generate a complete three-dimensional representation, a volumetric matrix was constructed for each OCT volume by sequentially arranging the available B-scans according to their acquisition spacing. The distance between adjacent B-scans ranged from 30 µm (high-resolution protocol) to 240 µm (high-speed protocol), corresponding to approximately 3–11 unoccupied columns between adjacent slices in the lateral view.

The classic ResUNet model was trained on both healthy and pathological OCT images from a publicly available dataset [17], encompassing various retinal conditions such as choroidal neovascularization (CNV), diabetic macular edema (DME), drusen, and normal retinal anatomy. Notably, ocular toxoplasmosis is not currently represented in any publicly available OCT dataset [18].

During reconstruction, the model predicted missing data to ensure spatial continuity across all B-scans within the volume. Training images were paired with synthetic masks containing randomly positioned vertical black stripes of varying thickness (1–10 pixels), simulating the lateral gaps present in OCT volumes. The model was subsequently fine-tuned on the current dataset, augmented to produce five variants of each B-scan, where the gap width varied within X ± 2 pixels (X = 3–11, depending on the acquisition protocol). This augmentation strategy improved the model’s ability to preserve the intrinsic volumetric characteristics of the retinal data. The study was conducted in accordance with the principles of the Declaration of Helsinki.

## 3. Results

This study included twelve eyes of twelve Caucasians patients (six women and six men). Patients’ baseline characteristics are summarized in Table 1. The mean (±) age was 39.2 years (range 14 to 67 years). The diagnosis and baseline evaluation were carried out within 16.2 days (min 5–max 49 days) of the onset of symptoms. Most common symptom was visual acuity decrease (50%), followed by myodesopsia (25%), blurred vision (16.7%), ocular pain (16.7%), and central scotoma (8.35%). TRC was primary in 84.4% and recurrent in 16.7%.

Only 5/12 patients were tested for Anti-Toxoplasma antibody: 5/5 were positive for IgG, 1/5 was positive for IgM, and 3/3 tested for PCR AC tap were positive for *T. gondii*. One patient tested positive for IgG *T. gondii*, negative for IgM *T. gondii*, and positive for PCR AC tap *T. gondii*.

The mean VA at baseline was 20/40 (6/12 eyes), followed by ≤20/50 (3/12 eyes) and ≤20/200 (3/12 eyes). Patients were treated either with trimethoprim + sulfamethoxazole (11/12) or with sulfadiazine + pyrimethamine (1/12) associated with oral prednisone (0.5 mg/kg) once daily, for at least 4 weeks. In total, 3/12 patients also required intravitreal treatment with clindamycin.

**Figure 3:**
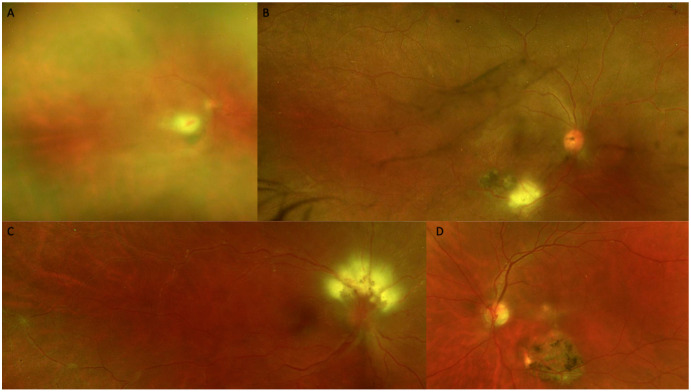
Color fundus photography (CFP) at the time of diagnosis. (**A**) Marked vitritis producing the classic appearance of a headlight in the fog. (**B**) An active white toxoplasmic retinochoroiditis lesion is visible adjacent to a retinal choroidal pigmented scar. (**C**) Optic nerve involvement is seen, vasculitis and a macular star. (**D**) Active macular lesion adjacent to a retinal choroidal atrophic scar.

Baseline biomicroscopic findings are summarized in Table 2 (see Figure 3).

**Figure 4:**
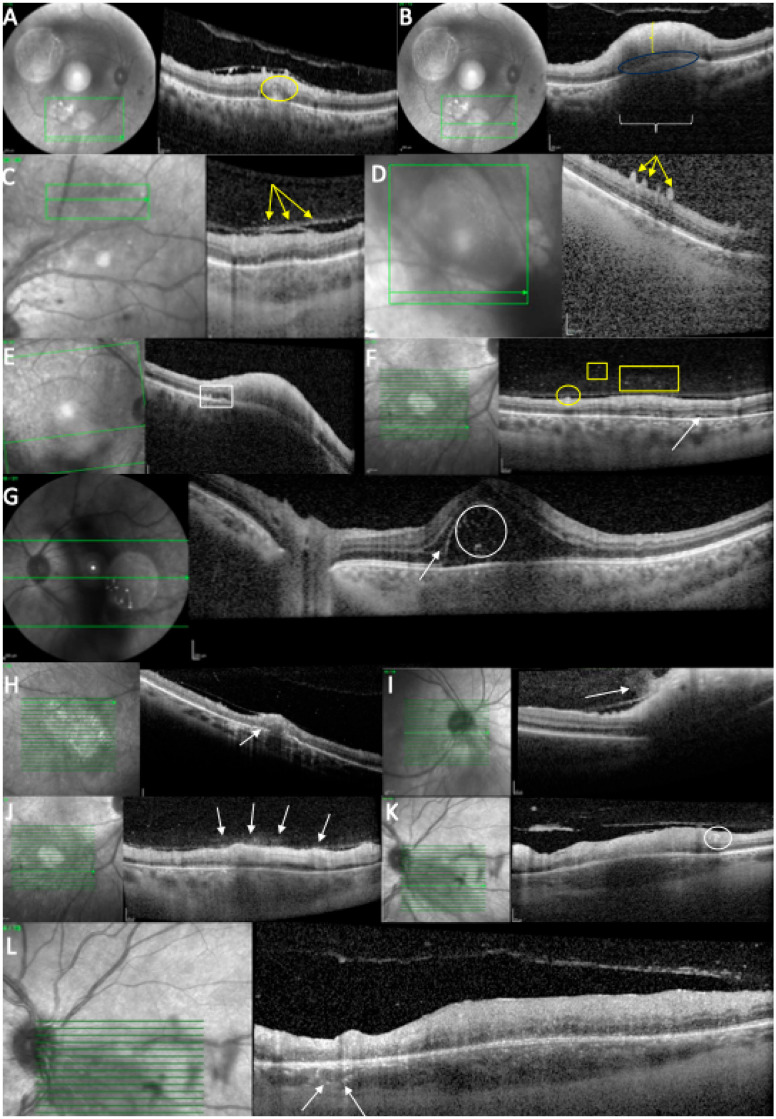
OCT scan at the time of diagnosis. (**A**) Intraretinal hyperreflectivities (circle) in the ONL, close to the TRC lesion. (**B**) Retinal hyperreflectivity (vertical bracket), increased thickness, RPE bowing (ellipse), and choroidal hyporeflectivity (horizontal bracket). (**C**) Posterior hyaloid hyperreflectivities (arrows). (**D**) ILM deposits (arrows). (**E**) RPE bumps (rectangle) and RPE bowing. (**F**) ILM deposits (circle), hyperreflective vitreous dots (square/rectangle), and subretinal fluid (arrow). (**G**) Subretinal hyperreflective dots (circle) and intraretinal fluid (arrow). (**H**) Increased RPE thickness (arrow). (**I**) “Hairy appearance” (arrow). (**J**) Hyperreflective dots above the blood vessels (arrows). (**K**) Hyperreflective dots around a retinal blood vessel (circle). (**L**) Hyperreflective dots around choroidal vessels (arrows).

At baseline, all cases (12/12 (100%)) demonstrated similar findings of the acute phase, including increased retinal thickness at the level of TRC lesion, and 11/12 (91.67%) cases showed full-thickness hyperreflectivity of the neurosensory retinal layers (see Table 3 and Figure 4). Mean retinal lesions thickness was 522.8 ± 293.5 μm. Disorganized retinal layers adjacent to lesion was a presenting feature in 10/12 (83.3%) lesions. Intraretinal OPL and ONL hyperreflective dots were a common presenting feature in 11/12 (91.67%) cases, adjacent to or far away from the active lesions. Periarteriolar hyperreflective dots were visible in 4/12 (33.3%) cases, while intraretinal fluid and ONL or OPL cysts were observed in 3/12 (25%) cases. Subretinal fluid was present in 4/12 (33.3%) eyes and subretinal hyperreflective dots in 2/12 (16.7%) eyes. Changes in RPE layer were observed as a thickened RPE in 6/12 (50%) cases, as RPE bumps in 2/12 (16.7%) cases, or as a bowing of retina–RPE–Bruch’s membrane in 7/12 (58.30%) cases. Macular hole was observed in 1/12 (8.35%) eyes. Liquefactive necrosis was already observed at baseline in 3/12 (25%) cases. The interval from symptom onset was 10, 14, and 49 days in these three patients. In terms of choroidal findings, 11/12 (91.67%) active lesions demonstrated significant choroidal thickening beneath the retinal active lesions at the acute phase, coupled with hyporeflectivity of the choroid in 11/12 (91.67%) eyes. Hyperreflective dots around choroidal vessels were also noted in 4/12 (33.3%) eyes. Mean choroidal thickness was 514.1 μm, but due to increased retinal hyperreflectivity, choroidoscleral interface was visible only in 8/12 eyes.

Vitreous SD-OCT findings included hyperreflective vitreous dots in 8/12 (66.66%) eyes. In addition, posterior hyaloid thickening was noted in 8/12 (66.66%) eyes. Hyperreflective vitreous dots were also observed over a blood vessel in 3/12 (25%) eyes while hyperreflective deposits were noted over the ILM in 11/12 (91.67%) eyes or on the posterior hyaloid in 8/12 cases (66.66%).

All the included patients have been consulted and OCT-imaged at follow-up (see Table 4 and Table 5). The mean BCVA at follow-up was 20/40 (9/12 eyes), followed by ≤20/50 (1/12 eyes) and ≤20/200 (2/12 eyes). Only 4/12 (33.3%) still exhibited thickened retina despite 12/12 (100%) eyes displaying full-thickness retinal hyperreflectivity. Intraretinal OPL and ONL hyperreflective dots were still observed in 5/12 (41.67%) cases, while periarteriolar hyperreflective dots, subretinal fluid, subretinal hyperreflective dots, thickened RPE, or RPE bumps were no longer detectable on follow-up OCT scans. Disorganized retinal layers adjacent to lesions were found in 12/12 cases (100%). The RPE layer exhibited atrophy and hypertransmission in most of the cases (9/12 (75%)). In terms of retinal destruction, two patterns were identified: the most common was liquefactive retinal necrosis, described as hyporeflective spaces or signal voids within the retina, and preservation of the RPE and ILM in 8/12 (66.66%) cases. Less commonly, coagulative retinal necrosis was found in 4/12 (33.3%) cases, previously described as full-thickness retinal hyperreflectivity and thickening. Over time, this type of retinal destruction progressively becomes thinner, less organized, and less hyperreflective.

In terms of choroidal changes, choroidal hyporeflectivity was still found in 9/12 (75%) cases, with choroidal thickening in 4/12 (33.3%). Hyperreflective dots were noted around choroidal vessels in 4/12 (33.3%) cases or in the choroid in 9/12 (75%). SD-OCT demonstrated posterior hyaloid thickening in 5/12 (41.67%) eyes. In 5/12 (41.67%) eyes, hyperreflective vitreous dots were identified; in 7/12 (58.30%) eyes, hyperreflective ILM deposits; and in 6/12 (50%) eyes, hyperreflective deposits on the posterior hyaloid. Choroidal thickness under the lesion at follow-up was 261.1 μm ±111.3.

Vitreous SD-OCT findings visible at follow-up included hyperreflective vitreous dots in 5/12 (41.16%) eyes. In addition, posterior hyaloid thickening was noted in 5/12 (41.16%) eyes. None of the eyes presented hyperreflective vitreous dots over a blood vessel while the hyperreflective deposits were noted over the ILM in 7/12 (58.30%) eyes or on the posterior hyaloid in 6/12 cases (50%). Incomplete PVD was observed in 7/12 (58.30%) eyes.

**Figure 5:**
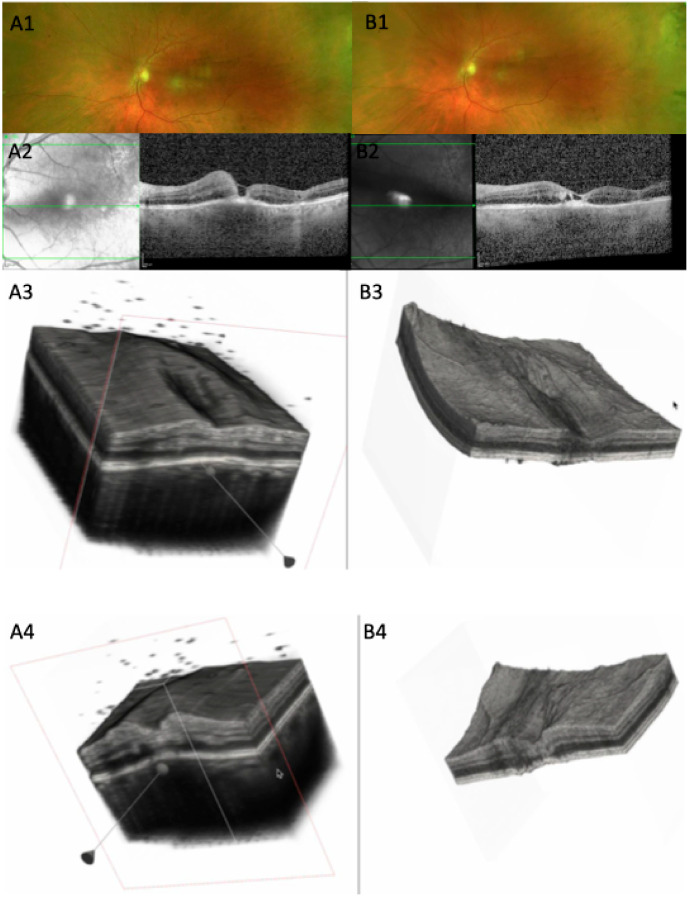
(**A1**) CFP at baseline, 10 days after symptom onset: white chorioretinal foveal lesion. (**A2**) OCT scan: parafoveal increased retinal thickness, with macular hole, increased RPE thickness, RPE bowing. (**A3**) Three-dimensional reconstruction showing increased retinal thickness around the fovea, with vitreous cells, thickened choroid, and RPE. (**A4**) Cross-section of the 3D model; increased retinal thickness is visualized at the edge of the fovea; dispersed vitreous cells. (**B1**) CFP 12 days from baseline, with retinal lesion starting to fade. (**B2**) OCT of the same horizontal slab as A2 showing decreased retinal thickness, multiple hyporreflective spaces, and almost flat RPE line. (**B3**) Large area surrounding the fovea of decreased retinal thickness showing irregular retinal surface. (**B4**) Section through the lesion showing altered retinal thickness with gliotic hyperreflective retinal tissue and less RPE bowing (see Supplementary Video S1).

**Figure 6:**
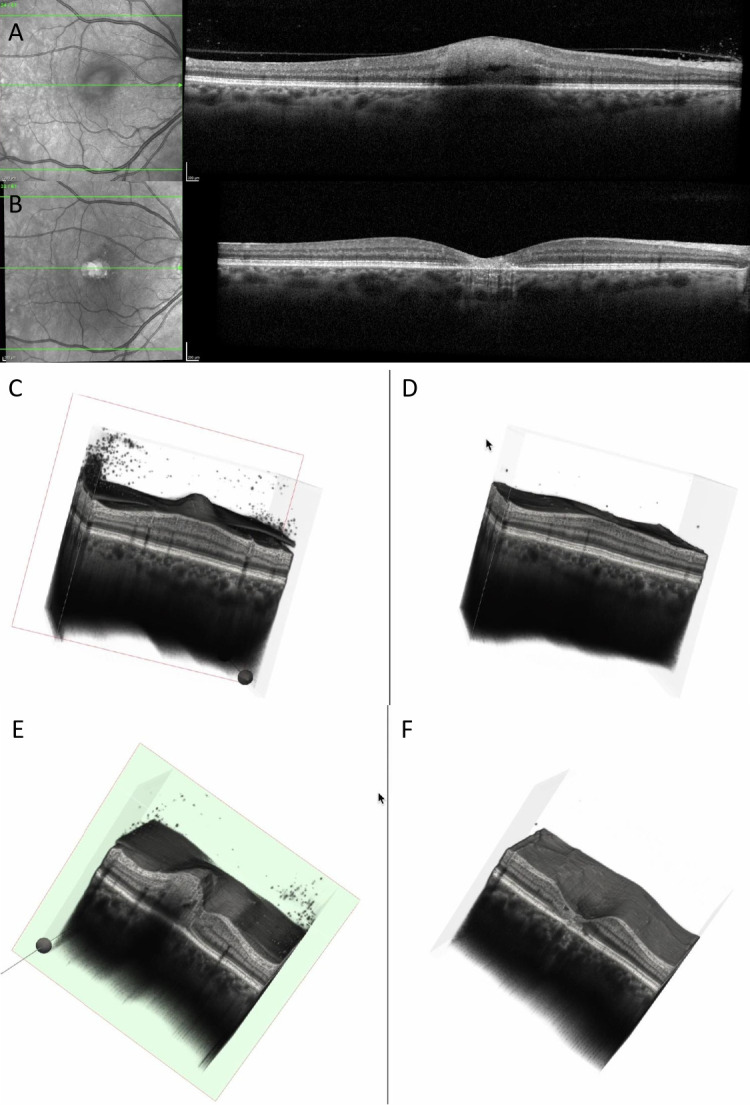
(**A**) OCT at baseline showing increased and hyperreflective retinal lesion, with a hyporeflective space. (**B**) OCT at 6 months showing decreased retinal thickness, hyperreflective gliotic tissue, discontinuity of RPE, EZ, ELM, and choroidal hypertransmission. (**C**) Three-dimensional reconstruction at baseline showing perifoveal increased retinal thickness, with vitreous cells and attached posterior hyaloid. (**D**) Three-dimensional reconstruction at 6 months showing enlarged foveal depression, disappearance of vitreous cells, and posterior vitreous detachment. (**E**) Cross-section of the 3D model at baseline showing increased perifoveal retinal thickness, choroidal shadowing, dispersed vitreous cells, and intraretinal hyporeflective spaces. (**F**) Cross-section of the 3D model at 6 months showing enlarged foveal depression with discontinuity of RPE, EZ, and ELM; the retinal vessels are prominent on the surface of the retina (see Supplementary Video S2).

**Figure 7:**
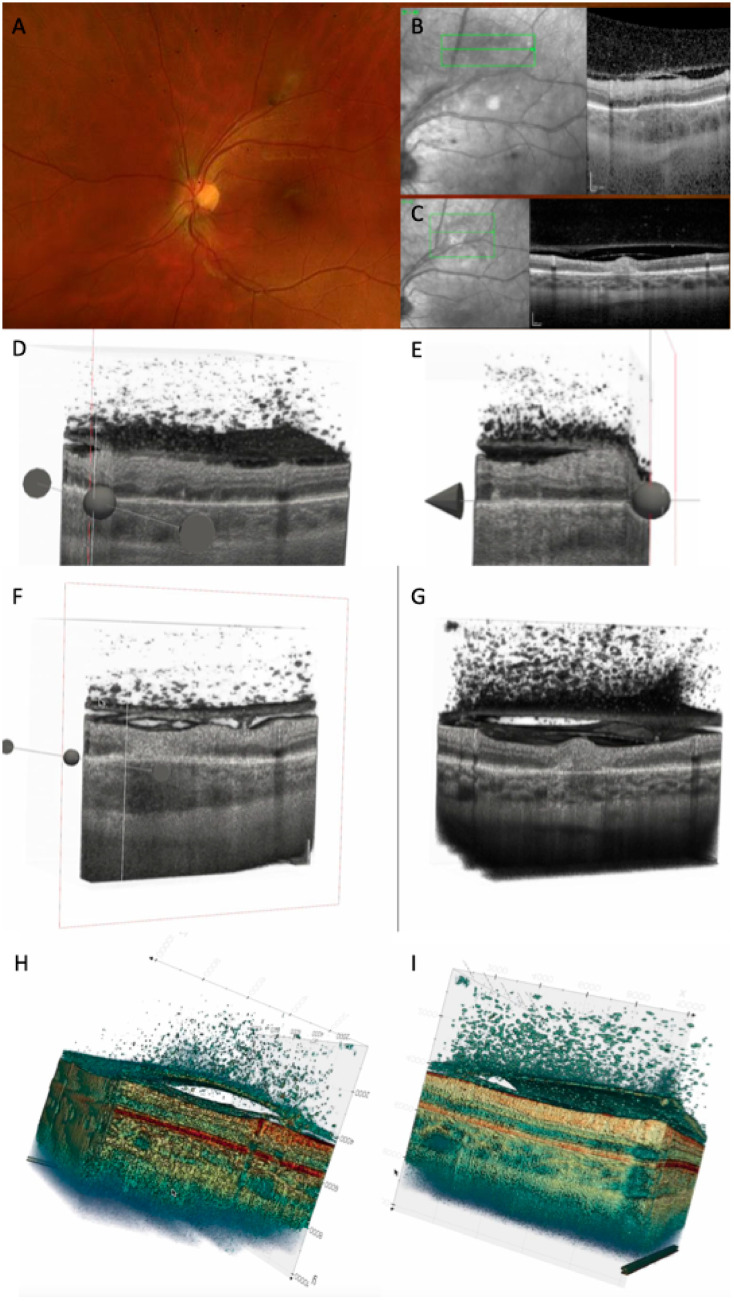
(**A**) CFP 35 days after onset showing TRC along the vascular arcade. (**B**) OCT scan at baseline showing retinal hyperreflectivity with increased choroidal thickness underneath, thickened posterior hyaloid with hyperreflective cells in the vitreous and on the hyaloid, and thickened and partially detached ILM with hypereflective cells. (**C**) OCT scan after 35 days: decreased retinal thickness around the lesion was noted, as well as decreased choroidal thickness and hyporeflectivity, and increased detachment of posterior hyaloid. (**D**) Three-dimensional reconstruction at baseline: numerous vitreous cells are depicted; attached posterior hyaloid, RPE bumps, and ONL hyperreflectivities are observed. (**E**) Three-dimensional reconstruction after 35 days: partially attached posterior hyaloid with persistent vitreous cells. (**F**) Cross-section of the 3D model at baseline showing increased choroidal thickness, adhesion between ILM and posterior hyaloid, and focal adhesion of hyaloid on the retinal surface. (**G**) Cross-section of the 3D model after 35 days, showing increased hyperreflective retinal lesion, with disorganized retina around it, decreased choroidal thickness, and increased PVD. (**H**) Three-dimensional colored reconstruction at baseline and (**I**) after 35 days, better highlighting the distribution of hyperreflective dots: on the external face of ILM, between ILM and posterior hyaloid face, attached to the posterior hyaloid externa land internal face, and in the vitreous (see Supplementary Video S3).

**Figure 8:**
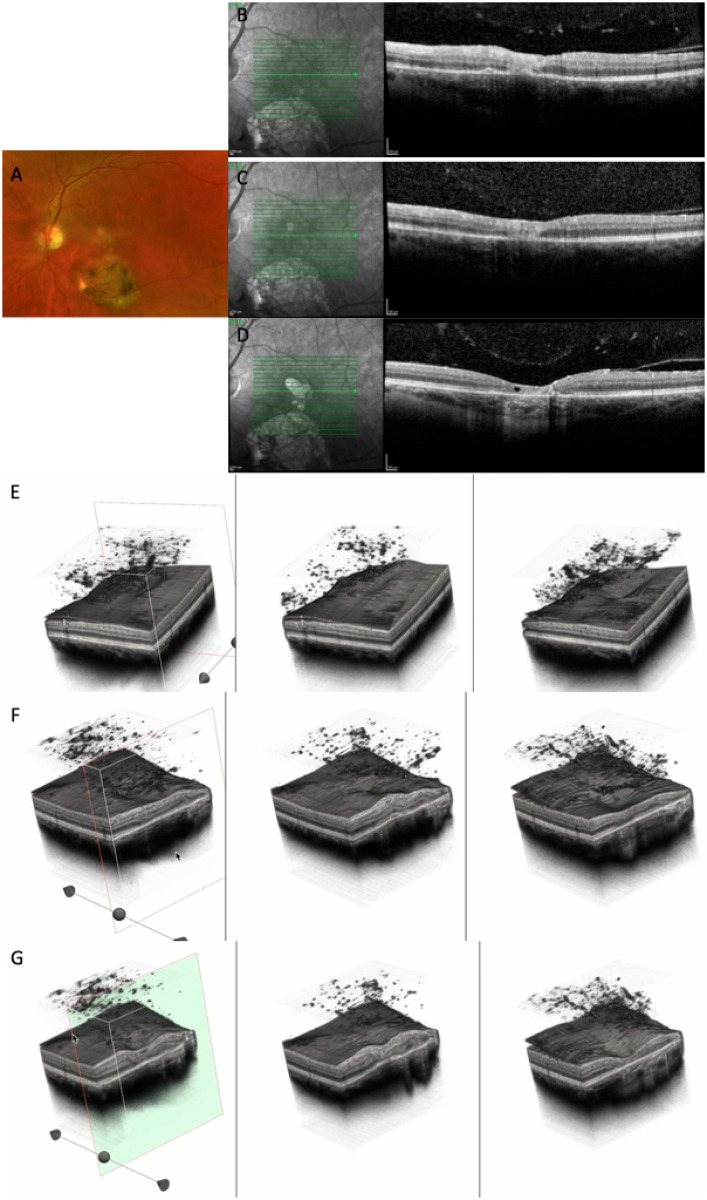
(**A**) CFP at baseline highlighting a large chorioretinal scar and an active perifoveal TRC. (**B**–**D**) OCT scans at baseline, at 10 days, and at 24 months, showing the transformation of an active foveal lesion into a chorioretinal scar. (**E**,**F**) Three-dimensional reconstruction of the entire retinal volume showing the dynamic transformation of the retinal architecture from an increased foveal thickness into a chorioretinal scar, together with increased choroidal transmission and the progression of the PVD. (**G**) Cross-section of the 3D model through the fovea depicting the transition from increased retinal thickness and hyperreflectivity to atrophy and gliotic tissue (see Supplementary Video S4).

**Figure 9:**
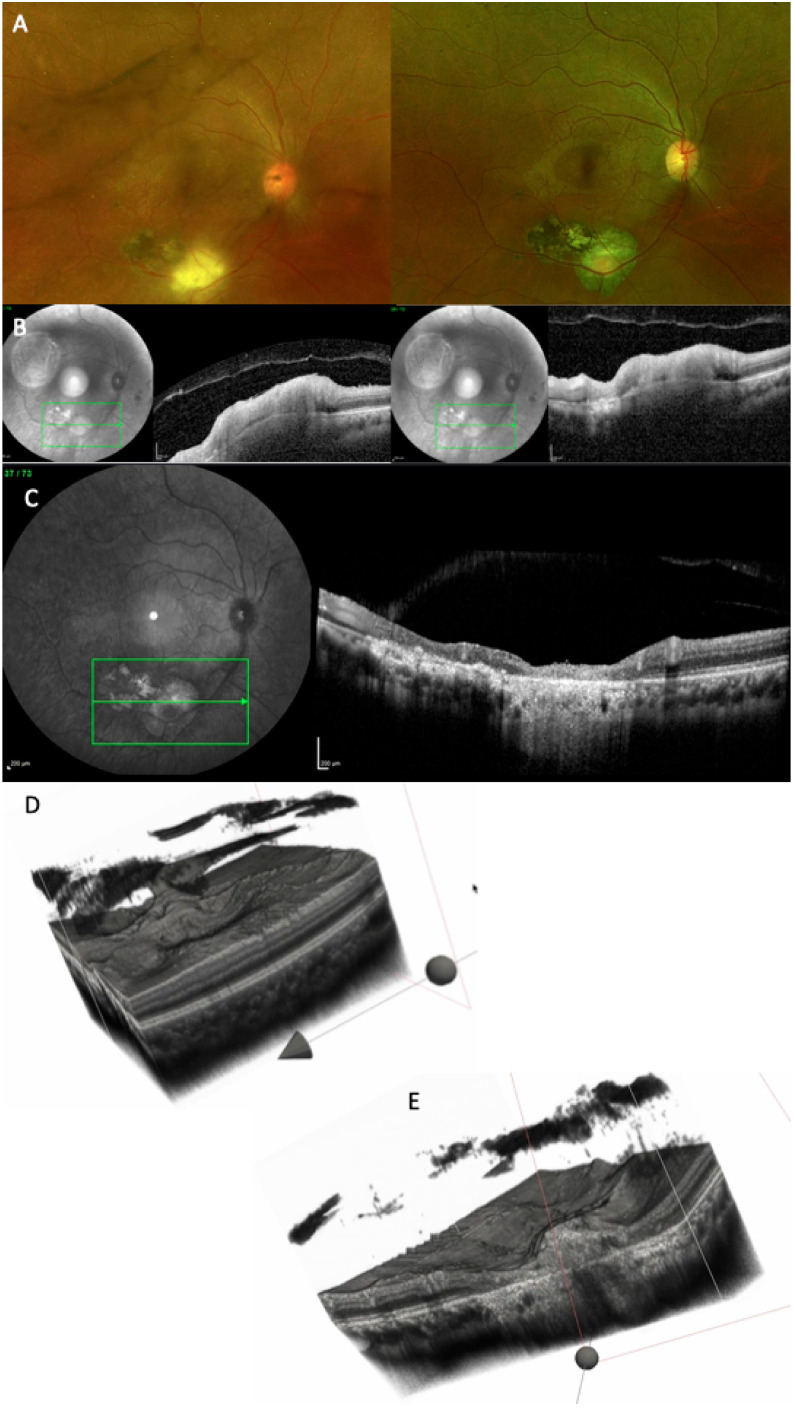
(**A**) CFP at baseline (left side) and at the 11-month follow-up (right side). (**B**) OCT scan at baseline: because the orientation of the images is different, the reconstruction could not be performed. (**C**) OCT scan at the 11-month follow-up, showing extensive chorioretinal damage, with merging of the old scar with the new one. (**D**) Three-dimensional reconstruction of the entire volume at baseline showing extremely irregular retinal surface and incomplete PVD. (**E**) Cross-section of the 3D model, depicting chorioretinal atrophy, gliotic tissue, RPE atrophy, and increased choroidal transmission (see Supplementary Video S5).

Several representative cases will be presented to illustrate the dynamic evolution of TRC lesions as visualized on OCT imaging (see Figure 5, Figure 6, Figure 7, Figure 8, and Figure 9). Following the three-dimensional reconstruction, we observed several characteristics of the retinal architecture during and after the acute infectious and inflammatory episode. Due to variations in orientation among the OCT sections, it was not possible to reconstruct all datasets. Only those sections with consistent orientation and adequate image quality were included in the three-dimensional reconstruction.

## 4. Discussion

The diagnosis of ocular toxoplasmosis is often based on characteristic clinical findings, as most patients present with classic features of a chorioretinal scar with a satellite lesion, and an area of active retinochoroiditis. However, OCT has become an invaluable tool in identifying and differentiating the various stages of the disease, allowing for a more detailed assessment of the retinal and choroidal structural changes throughout its evolution.

In the active necrotizing retinitis initial phase, *T. gondii* tachyzoites proliferate within the retina. This lesion will expand centrifugally forming a white fluffy area with indistinct margins [19,20]. Tachyzoites and inflammatory cells spread laterally, forming a lesion with a completely necrotic center and an edematous, actively inflamed periphery [21]. Due to immune response or treatment, tachyzoites convert into bradyzoites, forming tissue cysts within the retina [2,22]. The central necrotic area will stop expanding, and the inflammatory activity will decline from the center outward, showing a centripetal resolution. Our 3D reconstruction allowed us to spatially visualize the active TRC as a convex elevated lesion.

In our series, the most frequent OCT features observed during the active phase of TRC included a thickened, hyperreflective retinal lesion with multiple hyperreflective dots within the ONL and OPL, and marked disorganization of retinal architecture in the surrounding tissue. Additional findings consisted of a hyporeflective choroid beneath the lesion, focal thickening of the choroid, and the presence of hyperreflective deposits along the ILM. These changes were often accompanied by vitreous inflammatory cells located either above the lesion or along the retinal vessels, consistent with active intraocular inflammation. These findings align with previous reports describing significant inner retinal involvement during the acute phase [7,8,11,22]. Choroidal thickening and RPE bowing were clearly visualized on our 3D model.

During the healing phase, the retina becomes thinner and less hyperreflective, showing retinal pigment epithelium hyperreflectivity and excavation of varying severity but never with restoration of the normal architecture [23]. The necrotic retina is progressively replaced by fibroglial and pigment epithelial tissue, resulting in a sharply demarcated atrophic scar. The RPE migrates and proliferates over the lesion, producing pigment clumping at the margins. The scar becomes depigmented centrally with hyperpigmented borders due to RPE hyperplasia. The healed lesion manifests as a well-defined, atrophic chorioretinal scar, which could serve as a focus for recurrent reactivation, as dormant cysts can later release tachyzoites [19]. OCT appearance transitioned toward features of coagulative or liquefactive necrosis, with partial loss of retinal integrity and the emergence of a hyperreflective gliotic scar or atrophy of the RPE. The progressive decrease in choroidal reflectivity observed during this stage likely reflects the resolution of inflammatory infiltration and the gradual restoration of choroidal perfusion. The reduction in lesion thickness and reflectivity is consistent with the subsiding of active inflammation and tissue remodeling. The RPE could either become thicker, leading to choroidal hyporeflectivity, or show epithelial atrophy, leading to choroidal hyperreflectivity. Coagulative necrosis is characterized by preserved retinal tissue architecture. Conversely, liquefactive necrosis, seen as hyporeflective retinal spaces, is due to parasite proliferation and profound tissue destruction, as shown by Oliver, G.F et al. [7]. Different rates of OCT signs were reported by several authors, which could be partially explained by a different disease stage, or primary versus recurrent disease. In our population, changes involving the RPE were present as previously described, either as increased thickness, viewed as hyperreflectivity, or atrophy, seen as choroidal hypertransmission.

The OCT findings in both phases can be explained by the underlying immunopathology of *T. gondii* infection.

The hyperreflective retinal lesion seen in the active phase is presumed to correspond to intense inflammatory cell infiltration, edema, and necrosis associated with tachyzoite replication and the host immune response. However, this interpretation remains hypothetical, as no histopathological confirmation exists for infectious conditions. Hyperreflective spots can appear in a wide range of retinal diseases—infectious, inflammatory, and vascular—so their exact nature in toxoplasmosis cannot be definitively established.

Our assumption is supported only indirectly by prior studies suggesting that hyperreflective dots may represent inflammatory cells and could serve as biomarkers of inflammation. Their disappearance after treatment further supports this possibility but does not constitute proof. Frizziero et al. recently outlined four theoretical origins for hyperreflective spots on OCT: vascular elements (V-HRF), activated microglia (I-HRF), extravasated lipids or phagocytic cells (E-HRF), and RPE-derived cells (P-HRF) [24]. These categories highlight that multiple explanations are possible, and in our case the interpretation should be regarded strictly as a plausible hypothesis rather than a confirmed mechanism. Shadowing beneath the lesion reflects the dense inflammatory infiltrate and necrotic tissue that attenuate signal transmission. The hyporeflective or thickened choroid likely indicates choroidal vasculitis and secondary vascular congestion. As healing progresses, gliotic hyperreflectivity corresponds to fibrotic scarring and glial proliferation, while choroidal thinning and reduced reflectivity denote vascular dropout and the resolution of inflammation. On OCT imaging, the center of an active lesion shows full-thickness retinal disorganization and atrophy, whereas the edges appear hyperreflective and thickened, gradually regressing inward over time. The area of active versus scarred retinochoroiditis was further characterized by Duraffour et al., who reported mean optic disc areas of 1.32 ± 1.59 and 1.79 ± 2.36, respectively [25]. The mean ratio between scarred and active lesion areas was 1.36 (range = 0.54–2.18). The increase in lesion area was observed in all our cases where reconstruction was performed, explained by the perilesional retinal disorganization.

Although found in most of our patients, hyperreflective dots in the vitreous cavity are not pathognomonic for parasitic retinal necrosis, since they were also found in viral retinitis as shown by Oliver G.F. et al., representing either parasite cysts or clumps of inflammatory cells [7].

Periarteriolar hyperreflective dots could represent infiltrating inflammatory cells (e.g., neutrophils, macrophages, or lymphocytes) accumulating in the perivascular retina, activated microglia along retinal vessels, perivascular exudation, immune complex deposition, or even early vascular sheathing. Histopathological studies confirm the perivascular lymphocytic infiltration [26].

Anatomically and immunopathologically, the posterior hyaloid functions as a scaffold. In young and middle-aged individuals, posterior vitreous detachment (PVD) is often incomplete, leaving the hyaloid adherent to the internal limiting membrane (ILM). During retinal inflammation, inflammatory cells released from the affected retina migrate into the vitreous cavity [8]. These cells tend to move toward the vitreoretinal interface and adhere to the posterior hyaloid. Because the hyaloid forms a physical barrier, the cells accumulate on its outer surface. Chemotactic gradients originating from the retinal lesion facilitate this aggregation. Some inflammatory cells also settle directly on the ILM. The posterior hyaloid itself may become inflamed—termed hyalitis—which increases its adhesiveness to inflammatory cells and debris [27]. The accumulation of these cells can sometimes elevate the hyaloid, producing a hyperreflective membrane-like appearance on imaging. Incomplete vitreous liquefaction limits the diffusion of inflammatory cells, resulting in their localized clustering. Our tridimensional reconstruction allowed us to emphasize the presence of hyperreflective dots in all the locations: in the vitreous, on the ILM, under and above the posterior hyaloid, or between the ILM and posterior hyaloid. Three-dimensional reconstruction allowed us to visualize the progression of PVD either as an accentuation of the detachment or as a complete detachment.

Fluorescein angiography and ICGA have played a distinct role in the management of patients with toxoplasma retinochoroiditis, as demonstrated long ago by Brancato et al. [28]. Using fluorescein angiography (FA) in active TRC, lesions show early hypofluorescence followed by intense late hyperfluorescence with blurred margins, along with hyperfluorescence of the optic nerve head and vascular leakage. Although FA is not routinely used as a diagnostic test, its value becomes evident in situations such as vasculitis, vascular occlusion, macular edema, macular neovascular membranes, and optic nerve involvement. Atmaca LS et al. found that multiple hypofluorescent satellite dark dots (SDDs) could be observed in both active and inactive TRC [29]. In contrast, ICGA highlights SDDs, and active lesions appear hypofluorescent—an observation also reported by Auer C et al. [30].

Previous studies have successfully attempted OCT-based volumetric reconstructions in other infectious and inflammatory ocular diseases, such as in vitro imaging of parasites [31], choroidal tuberculoma [32], vitreomacular interface disorders [33], subretinal fluid in central serous chorioretinopathy [34], proliferative diabetic retinopathy [35], and foveal retinoschisis [36], as well as two cases of healed ocular toxoplasmosis using SD-OCT [23]. However, to our knowledge, the present study is the first to apply tridimensional reconstruction techniques to visualize and analyze the dynamic structural changes in toxoplasmic retinochoroiditis. The 3D reconstruction revealed a centripetal regression pattern of the lesion, indicating that the healing process proceeds from the periphery toward the center rather than uniformly across the lesion volume. This spatial insight adds a novel dimension to our understanding of lesion evolution and may have implications for monitoring therapeutic response.

As expected for a retrospective analysis, our study has several limitations. The sample size was inherently limited due to the exclusion criteria applied, including the requirement for at least two acquisitions at different time points. In many cases, the degree of initial vitritis precluded OCT imaging, which substantially reduced the pool of eligible patients, despite the database containing a much larger number of potential cases. Furthermore, the image processing and reconstruction workflow was not fully automated; layer isolation and segmentation required partial manual intervention. This labor-intensive process constrained the feasibility of including additional patients in the analysis. A more standardized and well-defined image acquisition protocol would have allowed for more accurate comparisons both between patients and in the same patient over time. However, the selected protocol was appropriate given the degree of vitritis observed; in cases with marked medium opacity, high-detail imaging protocols cannot be reliably obtained. Motion artifacts, vitritis, and shadowing occasionally obscured lesion boundaries, making accurate delineation challenging across all slices. Additionally, the absence of histopathologic correlation restricts definitive interpretation of certain OCT features. Furthermore, quantification of the lesion’s volume was not attempted due to a difficult delineation of the exact limit of the active TRC which would have been challenging. Other potential limitations could be image misalignment software interpolation errors or missing data in areas of full-thickness retinal loss. Ideally, our analysis would have relied solely on acquisitions with a high number of sections, which would have enabled a reconstruction more closely reflecting the true anatomical appearance and reduced the need for interpolation. Although this limitation can make visual comparisons more challenging, the quantitative assessment of the B-scans across the lesions remains valid, as our analysis accounted for the acquisition-specific parameters, including the spacing between sections.

A direct comparison between baseline—defined as the time of presentation and treatment initiation—and the 1-month or 3-month follow-up would have provided a more objective assessment of disease progression on OCT imaging. Nevertheless, follow-up intervals varied due to several practical factors, including patient availability, scheduling constraints, and the inconsistent availability of OCT images at each visit.

We acknowledge that a quantitative assessment of lesion size would have been valuable; however, this was not feasible given the current limitations of OCT technology and the reduced scan quality in patients with decreased vitreous transparency. These factors made precise volumetric measurements extremely challenging. At present, even when 3D reconstruction is successful, it allows only for a qualitative and dynamic evaluation of lesion evolution rather than a reliable quantitative analysis.

Future work should focus on improving lesion boundary demarcation and segmentation, distinguishing among infiltrative, necrotic, and exudative components. Automated or semi-automated volumetric analysis of TRC lesions could enable more objective quantification of disease activity. Integrating OCT angiography (OCTA) with 3D reconstruction may further elucidate microvascular remodeling during healing and help characterize perfusion changes within and around the lesion.

## 5. Conclusions

In summary, OCT and 3D reconstruction provide a complementary approach for characterizing the dynamic structural changes of toxoplasmic retinochoroiditis. By linking imaging biomarkers to the underlying immunopathological processes, these techniques enhance our understanding of disease activity, offer potential tools for monitoring treatment response, and may ultimately improve the clinical management of ocular toxoplasmosis. This study demonstrates that 3D OCT analysis provides a valuable framework for visualizing the dynamic structural changes in toxoplasmic retinochoroiditis, bridging imaging findings with underlying inflammatory processes.

## Figures and Tables

**Table 1:** Patient and disease baseline characteristics.

	Characteristics	Unit (%)
Sex	female	6/12 (50)
	male	6/12 (50)
Age	years	39.17 ± 17.69
Laterality	right eye	7/12 (58.30)
	left eye	5/12 (41.67)
Disease type	recurrent	2/12 (16.7)
	primary	10/12 (83.3)
Symptom duration until diagnosis	days	16.2
Symptoms	visual acuity decrease	6/12 (50)
	myodesopsia	3/12 (25)
	blurred vision	2/12 (16.7)
	central scotoma	1/12 (8.35)
	ocular pain	2/12 (16.7)
Baseline VA	≥20/40	6/12
	≤20/50	3/12
	≤20/200	3/12
Serology	*T. gondii* IgM-positive	1 (8.35)
	*T. gondii* IgG-positive	5 (41.67)
PCR AC tap	*T. gondii*-positive	3/12 (25)
Systemic treatment	trimethoprim + sulfamethoxazole	11/12
	sulfadiazine + pyrimethamine	1/12
	oral prednisone	9/12
Intravitreal treatment	clindamycin	3/12
Topical treatment	corticosteroid	3/12
	IOP-lowering drops	3/12

VA = visual acuity; *T. gondii* = *Toxoplasma gondii*; PCR = polymerase chain reaction; AC = anterior chamber; IOP = intraocular pressure.

**Table 2:** Baseline biomicroscopic findings.

Biomicroscopic Findings	Signs	Unit (%)
anterior segment	keratic precipitates	5/12 (41.67)
	Tyndall	5/12 (41.67)
vitreous	vitreous cells	11/12 (91.67)
	snowballs	1/12 (8.35)
posterior segment	ERM	1/12 (8.35)
	macular edema	1/12 (8.35)
	white retinal lesion	12/12 (100)
	chorioretinal scar	2/12 (16.7)
	associated vasculitis near the lesion	2/12 (16.7)
	hemorrhage near the lesion	2/12 (16.7)
	perilesional exudation	2/12 (16.7)
	macular star	1/12 (8.35)
	Kyrieleis arteritis	1/12 (8.35)
	optic disc edema	1/12 (8.35)
	lesion involving the optic disc	2/12 (16.7)
	macular hole	1/12 (8.35)

ERM = epiretinal membrane.

**Table 3:** OCT features at baseline.

SD-OCT Features at Baseline	Unit (%)
mean lesion height (μm) ± −SD	522.77 ± 293.5
**Retinal Lesion**	
thickened retina	12/12 (100)
full-thickness retinal hyperreflectivity	11/12 (91.67)
intraretinal OPL and ONL hyperreflective dots	11/12 (91.67)
periarteriolar hyperreflective dots	4/12 (33.3)
intraretinal fluid with ONL or OPL cysts	3/12 (25)
subretinal fluid	4/12 (33.3)
subretinal hyperreflective dots	2/12 (16.7)
disorganized retinal layers adjacent to lesion	10/12 (83.3)
macular hole	1/12 (8.35)
thickened RPE	6/12 (50)
RPE bumps	2/12 (16.7)
bowing of retina–RPE–Bruch’s membrane	7/12 (58.30)
liquefactive necrosis	3/12 (25)
**Choroid**	
choroidal hyporeflectivity	11/12 (91.67)
choroidal thickening	11/12 (91.67)
hyperreflective dots around choroidal vessels	4/12 (33.3)
choroidal thickness under the lesion (μm) ±SD	514.1 ± 267.9
**Vitreous**	
posterior hyaloid thickening	8/12 (66.66)
hyperreflective vitreous dots	8/12 (66.66)
hyperreflective vitreous dots over a blood vessel	3/12 (25)
hyperreflective ILM deposits	11/12 (91.67)
hyperreflective deposits on the posterior hyaloid	8/12 (66.66)
incomplete PVD	4/12 (33.3)

OCT = optical coherence tomography; SD = standard deviation; OPL = outer plexiform layer, ONL = outer nuclear layer; RPE = retinal pigment epithelium; ILM = internal limiting membrane; PVD = posterior vitreous detachment.

**Table 4:** Follow-up biomicroscopic findings.

Biomicroscopic Findings	Signs	Unit (%)
anterior segment	keratic precipitates	2 (16.7)
	Tyndall	2 (16.7)
vitreous	vitreous cells	5 (41.67)
	snowballs	1 (8.35)
posterior segment	ERM	3 (25)
	macular edema	0
	chorioretinal scar	6 (50)
	associated vasculitis near the lesion	0
	hemorrhage near the lesion	0
	perilesional exudation	0
	macular star	0
	Kyrieleis	0
	optic disc edema	2 (16.7)
	lesion involving the optic disc	2 (16.7)
	macular hole	0

ERM = epiretinal membrane. Mean duration of follow-up was 144 days (min 12–max 490).

**Table 5:** SD-OCT features at follow-up.

SD-OCT Features at Follow-Up	Unit (%)
mean lesion height (μm) ±-SD	286.76 ± 163.3
**Retina**	
thickened retina	4/12 (33.3)
full-thickness retinal hyperreflectivity	12/12 (100)
intraretinal OPL and ONL hyperreflective dots	5/12 (41.67)
periarteriolar hyperreflective dots	0
intraretinal fluid with ONL or OPL cysts	2/12 (16.7)
subretinal fluid	0
subretinal hyperreflective dots	0
disorganized retinal layers adjacent to lesion	12/12 (100)
macular hole	1/12 (8.35)
thickened RPE	0
RPE bumps	0
bowing of retina–RPE–Bruch’s membrane	3/12 (25)
RPE atrophy and hypertransmission	9/12 (75)
liquefactive necrosis	8/12 (66.66)
coagulative necrosis	4/12 (33.3)
**Choroid**	
choroidal hyporeflectivity	9/12 (75)
choroidal thickening	4/12 (33.3)
hyperreflective dots around choroidal vessels	4/12 (33.3)
hyperreflective dots in the choroid	9/12 (75)
choroidal thickness under the lesion (μm) ± SD	261.1 ± 111.3
**Vitreous**	
posterior hyaloid thickening	5/12 (41.67)
hyperreflective vitreous dots	5/12 (41.67)
hyperreflective vitreous dots over a blood vessel	0
hyperreflective ILM deposits	7/12 (58.30)
hyperreflective deposits on the posterior hyaloid	6/12 (50)
incomplete PVD	7/12 (58.30)

SD = standard deviation; OPL = outer plexiform layer, ONL = outer nuclear layer; RPE = retinal pigment epithelium; ILM = internal limiting membrane; PVD = posterior vitreous detachment.

## Data Availability

The raw data supporting the conclusions of this article will be made available by the authors on request.

## Supplementary Materials

The following supporting information can be downloaded at: https://www.mdpi.com/article/10.3390/diagnostics15233091/s1, Video S1: 3D reconstruction for Figure 5; Video S2: 3D reconstruction for Figure 6; Video S3: 3D reconstruction for Figure 7; Video S4: 3D reconstruction for Figure 8; Video S5: 3D reconstruction for Figure 9.

## Abbreviations

The following abbreviations are used in this manuscript:

OCT	Optical coherence tomography
PCR	Polymerase chain reaction
SD	Standard deviation
OPL	Outer plexiform layer
ONL	Outer nuclear layer
RPE	Retinal pigment epithelium
ILM	Internal limiting membrane
PVD	Posterior vitreous detachment
ELM	External limiting membrane
ERM	Epiretinal membrane
VA	Visual acuity
*T. gondii*	*Toxoplasma gondii*
AC	Anterior chamber
IgM	Immunoglobulin M
IgG	Immunoglobulin G
EZ	Ellipsoid zone
TRC	Toxoplasmic retinal choroiditis
CFP	Color fundus photography
AMD	Age-related macular degeneration
3D	Three-dimensional
